# Exploring temporal activity of dholes, their prey, and competitors in East Java, Indonesia

**DOI:** 10.1002/ece3.11666

**Published:** 2024-07-04

**Authors:** Linnea Worsøe Havmøller, Hariyawan Agung Wahyudi, Mochammad Iqbal, Ventie Angelia Nawangsari, Johan Setiawan, Desy Satya Chandradewi, Peter Rask Møller, Carl Træholt, Rasmus Worsøe Havmøller

**Affiliations:** ^1^ Natural History Museum of Denmark University of Copenhagen Copenhagen Denmark; ^2^ Research and Conservation, Copenhagen Zoo Frederiksberg Denmark; ^3^ Department for the Ecology of Animal Societies Max Planck Institute of Animal Behavior Constance Germany; ^4^ Copenhagen Zoo Baluran Programme, JI Raya Banyuwangi‐Situbondo Desa Wonorejo Indonesia; ^5^ Baluran National Park, JI Raya Banyuwangi‐Situbondo Desa Wonorejo Indonesia; ^6^ Directorate of Biodiversity Conservation of Species and Genetics Jakarta Indonesia; ^7^ Norwegian College of Fishery Science UiT – the Arctic University of Norway Tromsø Norway

**Keywords:** Baluran national park, camera trap, *Cuon alpinus*, *Panthera pardus*, Southeast Asia

## Abstract

Dholes (*Cuon alpinus*) are endangered large carnivores found in scattered populations in Asia. One of the main threats to dholes is the decreasing prey availability throughout their distribution range. In the present study, we used camera trap data collected over 6 years to investigate the temporal activity patterns of dholes and their putative prey species in Baluran National Park in Java, Indonesia. We also explored the overlap in activity between dholes and the park's other remaining large carnivore the Javan leopard (*Panthera pardus melas*), as well as humans. Furthermore, we investigated potential differences in activity patterns between dholes in packs and dholes roaming in pairs or alone. We found a high temporal overlap between dholes and their wild ungulate prey species (ranging from Δ = 0.66–0.90), with the lowest overlap observed between dholes and bantengs (*Bos javanicus*) (Δ = 0.66), and the highest between dholes and muntjacs (*Muntiacus muntjak*) (Δ = 0.90). A very low overlap was found between dholes and domestic cattle (*Bos indicus*) (Δ = 0.27) whereas a moderately high overlap was found between dholes and leopards (Δ = 0.70) and dholes and humans (Δ = 0.62). We found a significant difference in activity patterns between dholes in packs and dholes roaming alone or in pairs (Δ = 0.78, *p* = .01). Single/pairs of dholes were more active both during the day and at night, whereas packs were predominantly active around sunrise and sunset. The high overlap with humans potentially has a negative effect on dhole activity, particularly for dispersing individuals, and the low overlap with domestic species questions the extent to which dholes are considered to predate on them.

## Graphical abstract

Study of activity patterns and temporal overlap between dholes, their putative prey, sympatric predator and humans. In addition, we investigated differences in activity patterns of dholes in packs versus dholes roaming alone or in pairs.

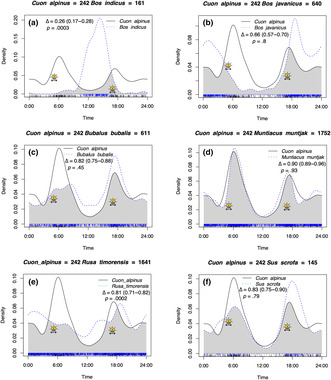

## 1 INTRODUCTION

Large carnivores and many of their prey species are in decline worldwide (Ripple et al., 2014; Wolf & Ripple, 2016). Understanding how predators and their prey interact is important, because it can reveal details about species dynamics that can be used to institute better management in the future. Assessing predator and prey activity patterns, and temporal partitioning between sympatric carnivores are important for making management decisions and conservation interventions especially in a world with increasing anthropogenic disturbance. According to foraging theory apex predators should favour prey with sufficient mass for optimal energy efficiency while at the same time minimise risk of injury (Stephens & Krebs, 1986). However, competition between sympatric carnivores may influence prey preference due to risk of kleptoparasitism (Balme et al., 2017; Karanth & Sunquist, 2000) and niche differentiation through spatial or temporal avoidance (Dröge et al., 2017; Hayward & Slotow, 2009; Krag et al., 2023). The presence of humans may further alter these interspecific interactions between predator and prey e.g., through the human shield hypothesis (Berger, 2007; Shannon et al., 2014) and between competing carnivores in the carnivore guild if one better tolerates humans (Parsons et al., 2022; Seveque et al., 2020). Effective and efficient management interventions are important for remnant populations of endangered species, where the survival of every single individual is critical. Once the treats have been identified well‐enforced legislation and management strategies have the ability to support recovery of large carnivores (Ingeman et al., 2022), e.g., with Bengal tigers (*Panthera tigris*) in Nepal doubling in numbers in 12 years (Dahal et al., 2023).

Asiatic wild dogs or dholes (*Cuon alpinus*) are pack‐living canids weighing 10–20 kg found in an extremely wide range of habitats, from tropical forests and grasslands to alpine steppe over 3000 m altitude (Wilson et al., 2009). Dholes were once found across Asia but have disappeared from over 80% of their historical range in recent years (Kamler et al., 2015; Ripple et al., 2014). Globally only about 2000 adult dholes are estimated to survive in the wild (Kamler et al., 2015). Dholes have been observed to form large packs with up to 30 individuals and hunt as a group (Fox, 1984). Their collaborative hunting technique enable them to take down prey many times their own body‐size (Durbin et al., 2004) with large packs taking down larger prey than smaller packs (George et al., 2021). Larger packs are also able to defend themselves and even kill sympatric carnivores such as leopards (*Panthera pardus*) and tigers (Prater, 1965; Venkataraman, 1995).

The disappearance of its natural medium to large‐bodied prey, with more than 40% of their prey species considered threatened, is an important contributing factor that makes dholes one of the most seriously affected large carnivores (Wolf & Ripple, 2016). Today dhole pack sizes are rarely recorded to exceed 10 individuals (Durbin et al., 2004). Smaller pack sizes potentially further contribute to their decline as it may translate into lower hunting and breeding success (Bhandari et al., 2021). Continuous habitat loss across its range, combined with the disappearance of suitable prey, persecution, and potential disease transmission also threatens dholes with local and regional extinction (Durbin et al., 2004; Kamler et al., 2015). As a result, dhole populations are found in small sub‐populations that are often in isolation of each other (Kamler et al., 2015). Long‐distance movement and dispersal of dholes is largely unknown but studies of African wild dog (*Lycaon pictus*) reveals that they suffer high mortality rates when dispersing (Cozzi et al., 2020; Woodroffe et al., 2020).

Baluran National Park (NP), located in the north eastern corner of Java, holds the last remnant of the once vast and ancient Sundaland savannah (Iyengar et al., 2005; Sodhi et al., 2004). This unique habitat is one of the few places on Java where dholes are still found (Havmøller et al., 2022). Baluran NP is also one of only three protected areas on Java that still have a population of the endangered native cattle banteng (*Bos javanicus*) (Pudyatmoko, 2017), the critically endangered Javan leopard (*Panthera pardus melas*) (Wibisono et al., 2021) and has a naturalised population of water buffalo (*Bubalus bubalis*) that has been present for hundreds of years (Long, 2003). In addition, several thousand domestic cattle (*Bos indicus*) is herded in and out of Baluran NP for pastoral grazing on a daily basis which has been suggested to be a point of conflict between people and dholes (Nurvianto et al., 2016).

In the last few decades, camera traps have become increasingly popular for conservation management and behavioural ecology research of especially mammals (Burton et al., 2015; Caravaggi et al., 2017; Rowcliffe, 2017). For example, knowledge gained from camera trap studies have improved our ability to study secretive species under conditions where direct observations are difficult or impossible (Linkie & Ridout, 2011; van Schaik & Griffiths, 1996). Also, activity studies documented temporal overlap between predators and their putative prey (Havmøller et al., 2020; Linkie & Ridout, 2011; Widodo et al., 2022) and temporal partitioning between sympatric carnivores (Hayward & Slotow, 2009; Krag et al., 2023; Sunarto et al., 2015).

In the present study, we investigate the temporal overlap in activity patterns of dholes, their putative prey, and the sympatric carnivore the Javan leopard from camera trap data collected over 6 years from 2015 to 2020 in Baluran NP, Indonesia. We do this to gain an understanding of how these patterns compare to other studies, because Baluran as a savannah ecosystem is fundamentally different in habitat composition and its species composition to other sites with dholes in Southeast Asia. In addition, we also assess activity patterns of dholes in packs versus dholes roaming in pairs or alone to gain insight into hunting times and when potential dispersers are temporally active, because lone dholes are more vulnerable to other carnivores and humans. We expect to find a high degree of overlap between dholes and their prey, whereas we expect to see temporal partitioning between dholes and leopards.

## 2 METHODS

### 2.1 Ethics statement

This study was conducted under a Memorandum of Understanding (MoU) between the Ministry of Environment and Forestry of the Republic of Indonesia and Copenhagen Zoo signed 21st of February 2014 and renewed on the 3rd of March 2023. Data were collected using camera traps deployed in grid system with no bait, direct contact or interaction with animals.

### 2.2 Study site

**FIGURE 1:**
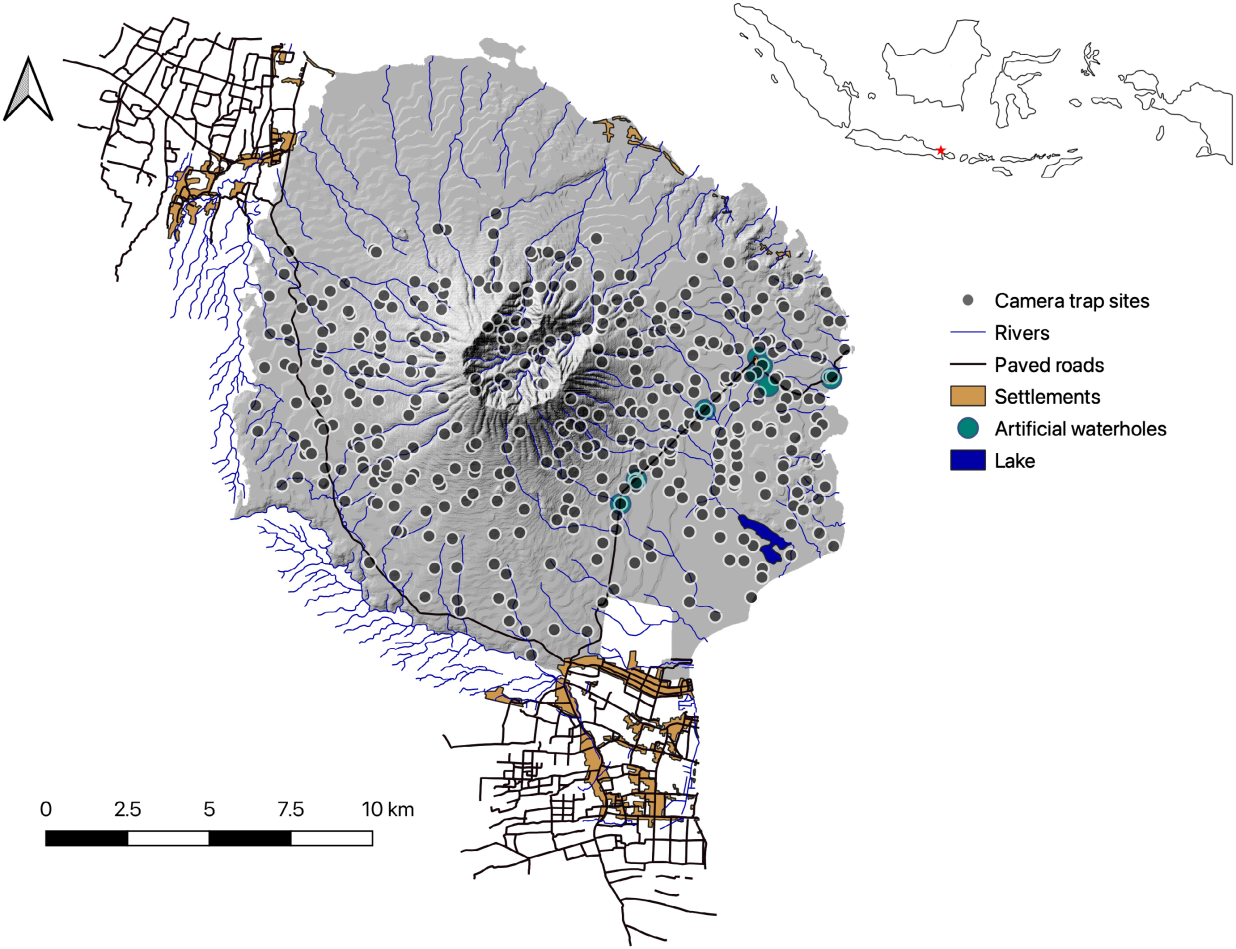
Map over Baluran National Park, location of Baluran National Park in insert top right marked by red asterisk with camera trap locations illustrated as black dots from 2015 to 2020.

Baluran National Park (NP) spans over 250 km^2^ and is situated in Northeast Java (74°9′52.3″ S, 114°23′15.4″ E), Indonesia (Figure 1). Mount Baluran is a dormant volcano in the centre of the park. The park contains a mosaic of habitats including mountainous rainforest, lowland evergreen forest, open deciduous forest, coastal mangrove forest, and one the largest remaining savannah landscape in Southeast Asia (Wahyudi and Nurman in prep.)—the last remnants of the once vast Sundaland savannah (Iyengar et al., 2005; Sodhi et al., 2004). Originally, open savannah and woodland savannah made up about half of the park but in the 1960s the alien invasive African acacia tree (*Acacia nilotica*) was introduced in surrounding areas as fire brakes (Pudyatmoko, 2019; Sutomo et al., 2020). Today, this species has spread to the entire park and overgrown more than 70% of the original savannah habitat (Sutomo et al., 2020). Bordering Baluran NP is the Bali Strait, teak plantations, villages, and a highly trafficked tarred road which cuts through the western part of the park (Figure 1). Baluran NP has two distinct seasons; a rainy season from November–March and dry season from April–October where the ephemeral water sources dry up and water is only available from a few small natural springs emerging from the volcano, or along the sea side as well as from artificial waterholes maintained by the National Park (Zoo, 2012). A settlement is situated inside the national park in the northern savannah (Figure 1) with inhabitants using the park for livestock grazing and crop plantation (Pudyatmoko, 2017; Pudyatmoko et al., 2018). In addition, various activities of tourism, non‐timber harvest, illegal logging and poaching has been documented (Nurvianto et al., 2015b).

### 2.3 Camera setup and analysis

Camera trapping was conducted from March–December in consecutive years 2015–2020. Camera traps were deployed in a grid system of 1 × 1 km covering a total area of 130 km^2^ with some grids used in multiple years (Figure 1). The camera traps used were Bestguarder (Bestguarder, China) and Covert (Covert Scouting Cameras, LLC, Russellville, KY). Cameras were installed approx. 30–45 cm from the ground with the camera position to an animal trail to monitor the mammal community at a distance of 2.5 m following (Ancrenaz et al., 2012). Cameras were set to record either video or photographs and checked approximately every 2 weeks.

Pictures were sorted and annotated following Sanderson and Harris (2013) and analysed in the software R v. 4.0.3 (R Development Core Team, 2020) using the packages ‘Activity’ v.1.3.3 (Rowcliffe, 2022) and ‘overlap’ v.0.3.4, (Meredith & Ridout, 2021). We used camera trap data for dholes, their putative prey, humans, and leopards. We identified eight prey species: domestic cattle, banteng, water buffalo, muntjac (*Muntiacus muntjac*), Timor deer (*Rusa timorensis*), Asian palm civet (*Paradoxurus hermaphroditus*), wild boar (*Sus scrofa*), and Javan langur (*Trachypithecus auratus*) known to be part of the diet of dholes (Hayward et al., 2014; Nurvianto et al., 2016) that also had sufficient data for analysis.

To assess if the sampled species had a random activity pattern over a circadian cycle the Hermans–Rasson test was performed (Landler et al., 2019). To estimate overlap in temporal activity patterns we followed Ridout and Linkie (2009), an interval of 30 min between images of the same species was used as independent events (Linkie & Ridout, 2011). To fit a non‐parametric circular kernel‐density function times extracted from photographs were converted to radians (Meredith & Ridout, 2021). The overlap coefficient (Δ) was used to calculate the overlap between the kernel‐density estimates of selected species, which ranges from no overlap (0) to complete overlap (1) (Linkie & Ridout, 2011; Ridout & Linkie, 2009). All species had >75 independent camera trap records hence the coefficient Δ4 was used in all instances (Meredith & Ridout, 2021). In order to compare the 24 h‐distribution records for each species pair a bootstrap test was conducted and confidence intervals were calculated as percentile intervals based on 10,000 bootstrap samples (Meredith & Ridout, 2014; Rowcliffe et al., 2008). A Wald test was used to test for significance of activity levels of pairwise comparisons, estimated using the ‘CompareAct’ function from the ‘activity’ package (Rowcliffe et al., 2014).

To test for difference in activity patterns between dholes roaming alone or in pair vs packs with three or more individuals, the same analysis was repeated. Dhole photos were therefore as packs if three or more dholes were seen within the same photo/video or if multiple individuals were observed going in the same direction with less than five minutes between photos (Chatterjee et al., 2021).

## 3 RESULTS

The total effort resulted in 24,544 camera trap days in which dholes accounted for a total of 242 independent detections (>30 min) in the survey period. Packs with at least three individuals were detected 87 times whereas dholes roaming alone or in pairs were detected 192 times. The largest identified dhole pack consisted of at least 13 adult individuals recorded by camera traps, and the mean pack size was 5.3 dholes (excluding pups). The most frequently detected species was humans (*Home sapiens*) with 6797 independent detections. The Hermans‐Rasson test was significant for all species tested, meaning all species exhibited non‐random activity patterns (Table 1).

**TABLE 1:** Number of independent events (*N*) separated by 30 min, and results from the Hermans–Rasson uniformity test of random activity pattern over a circadian cycle for dholes, their putative prey species, leopards, and humans derived from camera trap data from Baluran National Park, Java, Indonesia.

Scientific name	Common name	Hermans‐Rasson test	
		*N*	*p*‐value
*Cuon alpinus*	Dhole	242	.005
*Bos indicus*	Domestic cattle	161	<.001
*Bos javanicus*	Banteg	640	<.001
*Bubalus bubalis*	Water buffalo	611	<.001
*Homo sapiens*	Human	6797	<.001
*Muntiacus muntjak*	Muntjac	1752	<.001
*Panthera pardus*	Leopard	653	<.001
*Paradoxurus hermaphroditus*	Asian palm civet	192	<.001
*Rusa timorensis*	Timor deer	1641	<.001
*Sus scrofa*	Wild boar	145	.05
*Trachypithecus auratus*	Javan langur	89	<.001

**FIGURE 2:**
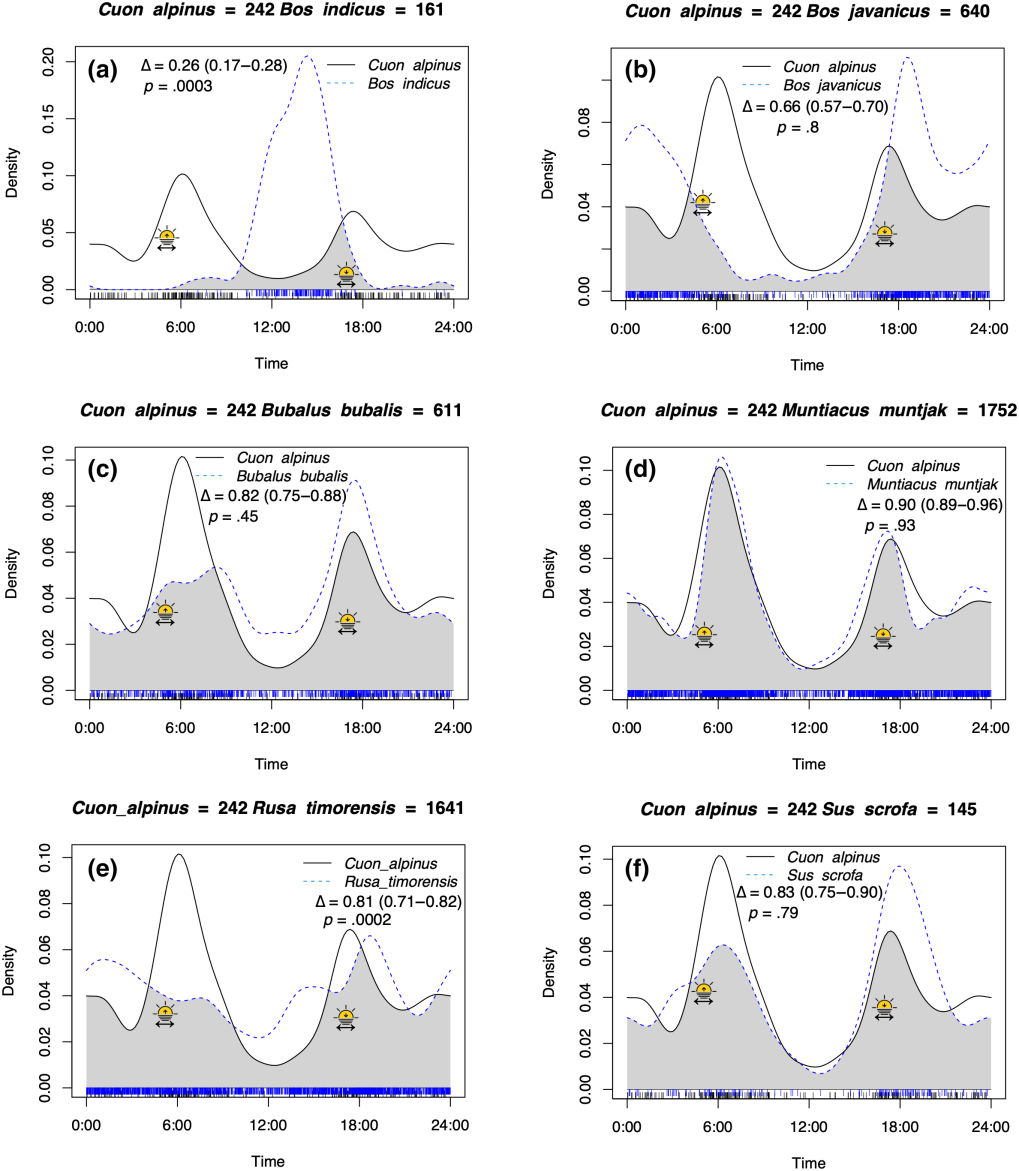
Activity patterns of dholes (*Cuon alpinus*) and overlap (grey area) with six species of ungulates known to be part of the diet of dholes in Baluran National Park, Java, Indonesia. Variation and time of sunrise and sunset is illustrated with a double‐sided arrow and a rising or descending sun in each species pair. (a) Domestic cattle (*Bos indicus*) was strongly diurnal with minimal overlap and significantly different activity pattern from dholes. (b) Banteng (*Bos javanicus*) was crepuscular with medium overlap. (c) Water buffalo (*Bubalus bubalis*) was crepuscular with high overlap. (d) Muntjac (*Muntiacus muntjac*) was crepuscular with very high overlap. (e) Timor deer (*Rusa timorensis*) was cathemeral with high overlap but exhibited a significantly different activity pattern. (f) Wild boar (*Sus scrofa*) was crepuscular with a high overlap. Included on each subfigure are their pairwise comparison overlap coefficients (Δ), 95% confidence intervals and *p*‐values.

**FIGURE 3:**
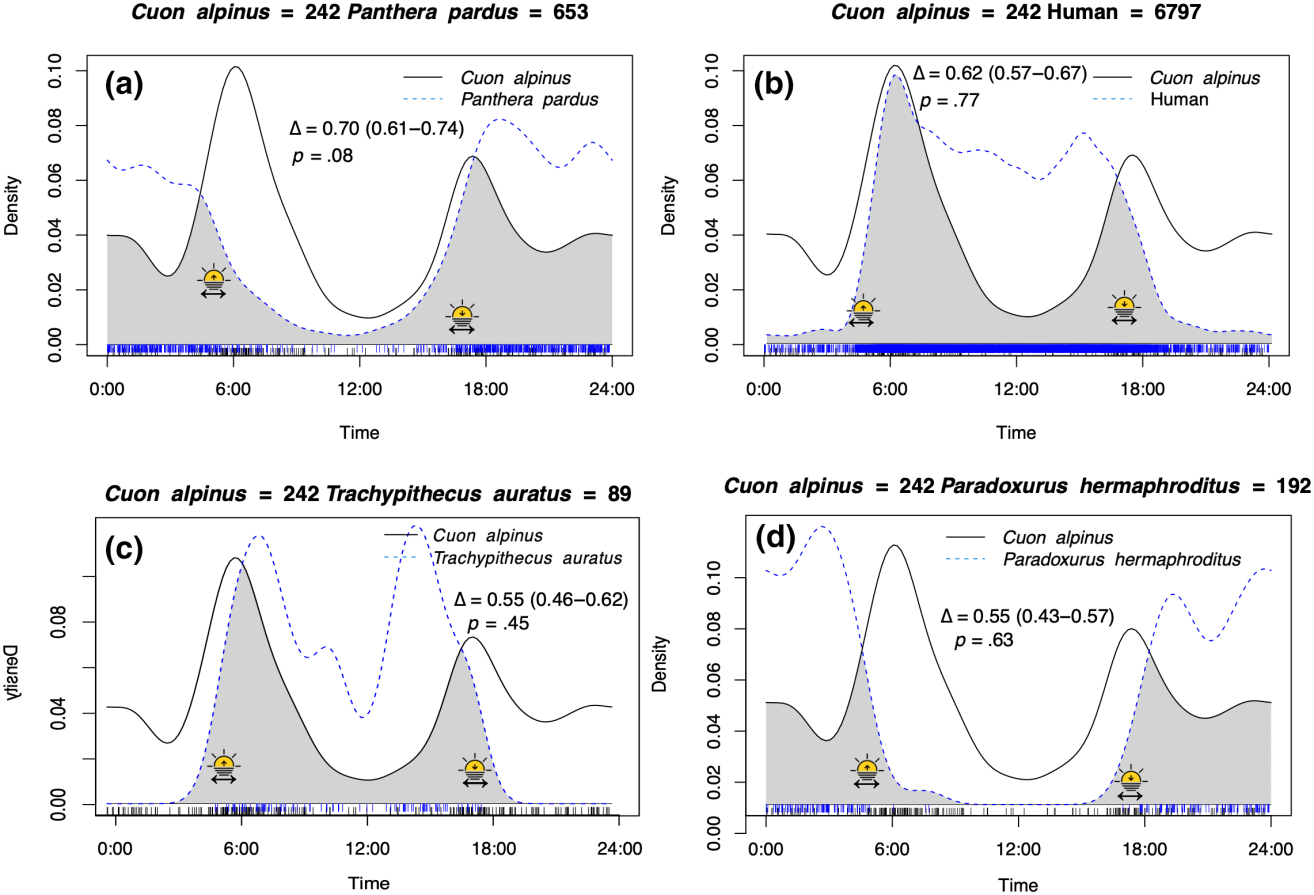
Activity patterns of dholes (*Cuon alpinus*) and overlap (grey area) with four species co‐occurring in Baluran National Park known to be competitors (a, b) or part of the diet of dholes (c, d). Variation and time of sunrise and sunset is illustrated with a double‐sided arrow and a rising or descending sun in each species pair. (a) Leopard (*Panthera pardus*) was unimodally nocturnal with a moderate temporal overlap. (b) Human (*Homo sapiens*) was strongly diurnal with medium overlap. (c) Javan langur (*Trachypithecus auratus*) was diurnal with medium overlap. (d) Asian palm civet (*Paradoxurus hermaphroditus*) was nocturnal and medium overlap. Included in each subfigure are the pairwise comparison overlap coefficients (Δ), 95% confidence intervals and *p*‐values.

Dholes exhibited a bimodal predominantly crepuscular activity pattern with peaks around sunrise and sunset with little activity during mid‐day (Figure 2). Six out of eight of the analysed prey species did not show a statistically different diel cycle compared to dholes, with only Timor deer and domestic cattle exhibiting significantly different activity patterns to that of dholes. Domestic cattle were diurnal peaking in the early afternoon (Figure 2a). Banteng exhibited a nocturnal activity pattern peaking shortly after sunset (Figure 2b). Water buffalo, muntjac, and wild boar displayed a crepuscular activity pattern though with a higher peak around sunset than sunrise (Figure 2c–f). Timor deer exhibited a cathemeral activity pattern with an almost uniform activity pattern throughout the diel cycle (Figure 2e). The Javan leopard exhibited a unimodal nocturnal activity pattern (Figure 3a). Human activity was high throughout daylight hours peaking around sunrise and early afternoon (Figure 3b). Javan langur exhibited a diurnal activity pattern with peaks in the morning and late afternoon (Figure 3c). Asian palm civet was strongly nocturnal (Figure 3e).

Temporal overlap coefficients between dholes, their prey, and competitors are listed in Table 2. The highest temporal overlap was found between dholes and muntjac with an overlap of 90% (CI: 0.89–0.96), followed by wild boar 83% overlap (CI: 0.75–0.90), water buffalo 82% (CI: 0.75–0.88), and Timor deer 81% (CI: 0.71–0.82). Overlap with banteng was moderately high at 66% (CI: 0.57–0.70) and a low overlap of 26% was found between dholes and domestic cattle (CI: 0.17–0.28). The temporal overlap with leopards was moderately high at 70% (CI: 0.61–0.74) and the overlap between dholes and humans was found to be 62% (CI: 0.57–0.67). A moderate overlap of 55% was found between both dholes and Javan langur (CI: 0.46–0.62) and Asian palm civet (CI: 0.43–0.57).

**TABLE 2:** Overlap coefficient (Δ) and 95% confidence interval (CI) between dholes, their prey and competitors in Baluran National Park, Java, Indonesia.

Scientific name	Common name	Overlap Δ	95% CI
*Bos indicus*	Domestic cattle	0.26^a^	0.17–0.28
*Bos javanicus*	Banteng	0.66	0.57–0.70
*Bubalus bubalis*	Water buffalo	0.82	0.75–0.88
*Homo sapiens*	Human	0.62	0.57–0.67
*Muntiacus muntjak*	Muntjac	0.90	0.89–0.96
*Panthera pardus*	Leopard	0.70	0.61–0.74
*Paradoxurus hermaphroditus*	Asian palm civet	0.55	0.43–0.57
*Rusa timorensis*	Timor deer	0.81^a^	0.71–0.82
*Sus scrofa*	Wild boar	0.83	0.75–0.90
*Trachypithecus auratus*	Javan langur	0.55	0.46–0.62

^a^ Represents significantly (*p* < .05) different activity patterns to dholes based on Wald test.

**FIGURE 4:**
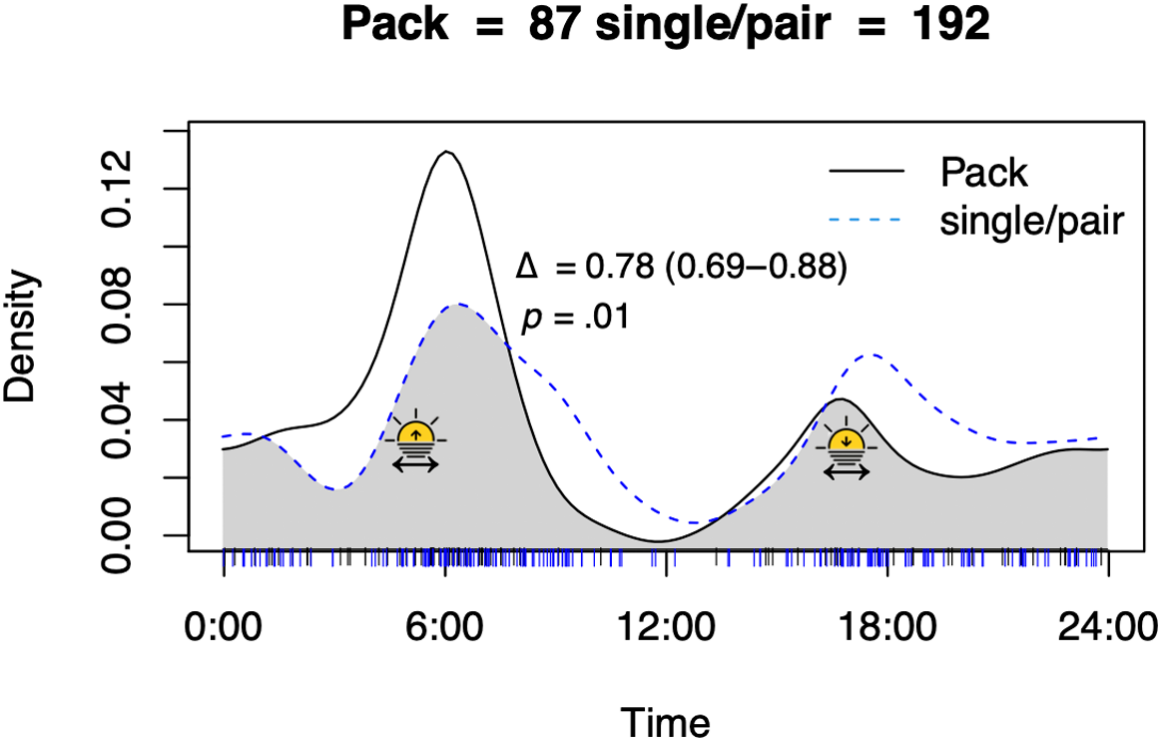
Activity patterns of dholes (*Cuon alpinus*) found in packs (consisting of min. three individuals) represented by a solid black line compared with dholes roaming alone or in pairs represented in a dotted blue line. The overlap is represented in grey, with overlap coefficient (Δ), 95% confidence interval and *p*‐value. Variation and time of sunrise and sunset is illustrated with a double‐sided arrow and a rising or descending sun in each species pair. Note that the activity of singles/pairs are prolonged subsequent to the peaks of packs.

We found a significant difference between activity patterns of dholes in packs vs single/pairs (Figure 4). Dholes roaming alone or in pairs were more active before noon than dholes roaming together in a pack of three or more individuals, additionally, single/pairs also peaked later at night than packs (Figure 4).

## 4 DISCUSSION

In our study, we found that dholes in Baluran NP exhibit a crepuscular activity pattern peaking in the morning around 6.00 and evening around 17.30 corresponding with the hour around sunrise and sunset (NOAA, 2023). Very little activity was found around mid‐day whereas dholes stayed moderately activity throughout the night. These findings are similar to dhole activity patterns observed in a previous study in Baluran NP (Pudyatmoko, 2019) and in Tadoba‐Andhari Tiger Reserve in central India (Ghaskadbi et al., 2016) but contrasting to many other study sites where dholes are mainly considered diurnal e.g., Northeast India (Bashir et al., 2014), South India (Karanth & Sunquist, 2000; Venkataraman et al., 1995), Malaysia (Kawanishi & Sunquist, 2008), Laos (Kamler et al., 2012), and Sumatra (Allen et al., 2020; Widodo et al., 2022). Even in Ujung Kulon in West Java dholes appear to be more diurnal although their activity peak around sunrise and sunset (Rahman et al., 2018). This could be caused by a combination of habitat type, climate, the composition of the carnivore guild, and human disturbance.

An open savannah likely alters the way and timing of dholes' hunts in comparison to a closed rainforest (Karanth & Sunquist, 2000). Ambient temperatures in Baluran NP can be as high as 37°C (Sutomo & van Etten, 2021) which could induce heat‐stress and impede dhole hunting capabilities during the hottest hours of the day. The extirpation of Javan tigers (*Panthera tigris sondaica*) in Baluran NP likely also changed the dynamic in the carnivore guild and may have led dholes to become increasingly crepuscular and targeting larger prey. We found that most of the putative prey species in Baluran NP are crepuscular or nocturnal (Figures 2 and 3) and predators are known to follow the activity patterns of their prey (Linkie & Ridout, 2011; O'Brien et al., 2003). However, predators may also change their activity pattern to temporally avoid competition (Havmøller et al., 2020), e.g., in southern India where tigers are present dholes appear to be more diurnal (Karanth et al., 2017; Karanth & Sunquist, 2000; Ramesh et al., 2012b) and the same has been observed in southern Thailand (Steinmetz et al., 2013). Lacking effective night vision is assumed to be one of the key reasons why African wild dogs are more diurnal than nocturnal (Rasmussen & Macdonald, 2012). It is possible that dholes have similar physiological limitations although this remains to be studied.

### 4.1 Temporal overlap with prey

Throughout their range dholes eat up to 50 different prey species (Srivathsa et al., 2020). In Baluran NP, Nurvianto et al. (2016) found 19 different vertebrate prey species in dhole scats, ranging from small rodents and tree shrews to water buffalo and banteng. As cursorial, pack hunters pursuing their prey we expected a high degree of overlap between dholes and their prey (Kawanishi & Sunquist, 2008). Furthermore, we expected their peak activity periods to correspond with hunting times, whereas leopards as ambush predators might have a lower level of overlap between peak activity periods and hunting behaviour (Havmøller et al., 2020). Johnsingh (1983) observed in Bandipur, India, that if a pack of dholes made a large kill in the morning they would only hunt once a day, whereas if the kill was not enough to sustain the whole pack they would go hunting again in the evening. This fits very well with our results, as we see a larger peak in activity in the morning than in the afternoon (Figure 2).

Following definitions by Lynam et al. (2013), we found a high >80% temporal overlap with the ungulates: muntjac, Timor deer, and wild boar (Figure 2). Our results suggest a much higher temporal overlap between dholes and their ungulate prey than a previous study in Baluran NP (Pudyatmoko, 2019). However, our study consists of a much larger sample size and over multiple years. We found a very high overlap (90%) between dholes and muntjac in Baluran NP. Muntjac is considered an important prey for dholes in e.g., Bhutan (Thinley et al., 2011; Wang & Macdonald, 2009), Thailand (Charaspet et al., 2020; Steinmetz et al., 2021), and Laos (Kamler et al., 2020) whereas muntjac is less often detected in dhole scats in India (Andheria et al., 2007; Ramesh et al., 2012a) and constituted <10% of the prey recorded in 54 dhole scats collected in Baluran NP (Nurvianto et al., 2016). According to Nurvianto et al. (2016), the species with the highest biomass contribution was water buffalo followed by banteng and Timor deer, water buffalo also had the highest frequency of occurrence. Pack size could influence prey preference with larger packs preferring larger prey as has been observed in India (George et al., 2021). However, there could also be seasonal variations in the diet of dholes in Baluran NP as has been observed in Laos and Bhutan (Kamler et al., 2020; Thinley et al., 2011). During the wet season the volcanic soil in the open savannah areas in Baluran NP become very muddy and impedes movement of both predators and prey. However, similarly to snow the mud might affect ungulates more than carnivores (Sullender et al., 2023) potentially making ungulates easier targets during the wet season.

We found a relatively high temporal overlap with the two larger bovids: water buffalo and banteng. Dholes have previously been suggested to supress banteng populations (Pudyatmoko et al., 2007; Venkataraman et al., 1995). To protect the endangered banteng, it was suggested to limit dhole pack sizes, as especially large packs of dholes, were believed to threaten the long‐term survival of banteng (Pudyatmoko et al., 2007). Banteng numbers in Baluran NP dramatically decreased around 2002 (Pudyatmoko et al., 2007), and it remains unknown to what degree dholes actively target banteng today, if they are hunted opportunistically or scavenged after poaching (Hayward et al., 2014; Nurvianto et al., 2016). Rahman et al. (2021) recently obtained photographic evidence of dhole predating on banteng in Ujung Kulon National Park, West Java. In both instances dholes killed or attempted to kill young banteng (Rahman et al., 2021). Pudyatmoko et al. (2007) observed dhole packs with up to 25 members and more in Baluran NP to take down banteng cows (some pregnant) and subadult males. During our study period we recorded camera trap videos of a pack of eight dholes harassing a mature banteng bull, and a pack of 13 dholes harassing a water buffalo at artificial water troughs. In both cases the bovines seemed annoyed by the dholes but continued to drink (Video S1), suggesting that a healthy adult bull does not have a high risk of predation even from a large pack of dholes.

A moderately high temporal overlap was found between dholes and Javan langur (55%) as well as between dholes and Asian palm civet (55%). Javan langurs are mainly arboreal and only rarely come down to the ground (Kool, 1993), dholes probably prey upon langurs opportunistically or scavenge (Johnsingh, 1983) but they do not appear to be part of dholes preferred prey (Hayward et al., 2014). According to the diet review by Hayward et al. (2014) from 16 locations including India, Bhutan, Laos, Malaysia and Thailand macaques (*Macaca* spp.) are more often eaten than langurs by dholes. However, crab‐eating macaques (*Macaca fascicularis*) were not found in dhole scats from Baluran NP (Nurvianto et al., 2016) and were therefore not included in our analysis. Asian palm civet was recorded in dhole scats in Baluran NP (Nurvianto et al., 2016), again this species is probably not part of the preferred prey (Hayward et al., 2014) but eaten opportunistically.

We found a low temporal overlap in activity between dholes and domestic cattle with only a 26% overlap in activity indicating they rarely overlap temporally. Domestic cattle were almost only detected in camera traps during the day when dholes were least active (Figure 2a), however the activity pattern observed for domestic cattle is heavily controlled by when herders release them into the park. Although domestic cattle has previously been observed in scats from dholes in Baluran NP (Nurvianto et al., 2016) this finding suggests that there is a narrow window for dholes to prey on livestock. To our knowledge, there has not been any reported retaliatory killings of dholes in Baluran NP due to livestock predation. However, retaliatory killing constitutes a major threat to dholes in other places within their distributional range e.g., in Bhutan, Nepal, and India (Aryal et al., 2015; Katel et al., 2015; Lyngdoh et al., 2014). It is also possible that dholes scavenge on domestic cattle that have died from other reasons.

### 4.2 Temporal overlap between sympatric carnivores

We found a moderate overlap in activity between dholes and leopards (70%), which is higher than previously detected from the same site but from a single season (39%) (Pudyatmoko, 2019). Although leopards exhibited a nocturnal activity pattern and dholes crepuscular the two carnivores did not have significantly different activity patterns. In a carnivore guild in the African savannah predators partition dietary niche in the prey community (Hayward & Kerley, 2008) and/or exhibit temporal niche partitioning to avoid competition (Hayward & Slotow, 2009; Krag et al., 2023). In Asia there appears to be a higher degree of overlap in prey consumed by sympatric carnivores (Hayward et al., 2014) and social dominance has been suggested to play a smaller role in shaping carnivore communities in tropical forests compared to the African savannah (Karanth & Sunquist, 2000). Baluran NP's large open savannahs make it unique to other carnivore systems in Southeast Asia. Dholes and leopards may compete more in open habitats where the prey biomass is comparatively higher than in i.e., rainforests (Cavada et al., 2019) but also more concentrated within a relatively small area. Dholes as cursorial hunters may favour these open areas for hunting whereas leopards as ambush predators have a higher success in denser cover (Karanth & Sunquist, 1995; Kawanishi & Sunquist, 2008; Ray‐Brambach et al., 2018). Dholes and leopards may instead spatially avoid each other although Pudyatmoko (2019) found the opposite. In India dhole hairs have been found in leopard scats suggesting leopards eat dholes (Karanth & Sunquist, 1995), but dholes in packs have also been observed to drive leopards into trees and kleptoparasite on their kills (Ramesh et al., 2012a; Venkataraman, 1995).

### 4.3 Human activity

We found that humans in Baluran NP were mainly active during the day with activity peaks around sunrise and early afternoon. These peaks overlapped with dhole peaks especially in the morning (Figure 3), indicating that dholes in Baluran NP do not temporally avoid humans. Human activity increased from almost no activity during the night to a very high activity within a short timeframe in the morning corresponding with the park opening. This probably means that humans are mainly concentrated near the park entrance and on the road going into the park, whereas dholes hunting are most likely found in other places of the park e.g., the savannah. Dholes might not temporally avoid humans but spatially avoid areas with high human disturbance, however, this needs further investigation. In the same study site Nurvianto et al. (2015b) found a lower probability of occupancy by dholes in areas with herding activity and non‐timber harvesting (Nurvianto et al., 2015b). Also in Baluran NP Pudyatmoko (2017) found a lower probability of occupancy by dholes and their prey in livestock grazing areas compared to areas not occupied by livestock, with similar results found in India (Milda et al., 2023; Srivathsa et al., 2014).

### 4.4 Packs versus singles/pairs

Not much research has focused on the social structure and dynamics of dhole packs (Bhandari et al., 2021) and it remains unknown if pack cohesion is similar to that of African wild dogs (Creel & Creel, 1995) or if they exhibit fission‐fusion dynamics as suggested by Prater (1965). We observed single or pairs of dholes to be both more diurnal and more nocturnal than packs consisting of at least three individuals. Although the overlap between pack and single/pair activity patterns were high with 78% overlap (CI: 0.69–0.88) (Figure 4), there was a significant difference between the observed activity patterns (*p* = .01). It is unclear whether these singles and pairs are temporal splits from larger packs, if they are dispersing individuals, or newly established pairs. A newly established pair will likely avoid larger packs bordering or overlapping in territories (Ghaskadbi et al., 2022). However, Baluran NP is a relatively small park with limited access to other protected areas which might drive newly stablished pairs to shift their activity pattern to limit competition rather than move spatially.

Dholes in Khao Ang Rue Nai Wildlife Sanctuary in Thailand were recorded by camera traps to be crepuscular here, no leopards and tigers were detected on camera traps and both species are thought to be extirpated (Jenks et al., 2012). A single dhole fitted with a GPS‐collared in Khao Ang Rue Nai Wildlife Sanctuary was found to be mainly diurnal (Jenks et al., 2015). Similarly, Nurvianto et al. (2015a) observed one radio‐collared dhole in Baluran NP to primarily be active in the hunting grounds during the light hours of the day or at moonlight nights. Based on activity patterns from camera traps at den sites feeding mainly took place in the early morning, afternoon and night with very little activity between 09:00 and 14:00 (Nurvianto et al., 2015a). It could be that dholes are more diurnal when roaming alone or in pairs as some might be returning to the den to check on pups, and/or dispersing individuals whereas the whole pack may be more crepuscular as dawn and dusk are the preferred hunting times (Johnsingh, 1983).

Dholes are communal hunters with extensive co‐operation although they have been observed occasionally to hunt alone or in pairs (Acharya, 2007; Pudyatmoko et al., 2007). All individuals do not always get their share of the meal if the kill is small which might lead to another hunt in the same day (Johnsingh, 1983). However, this might split the pack so only the hungry individuals attempt another hunt, which would fit well with our results that dholes in pair or alone have a higher, slightly delayed peak in activity in the evening (Figure 4).

Baluran NP consist of a very diverse habitat containing evergreen, mangrove, deciduous and alpine forest as well as large areas with open savannah. The habitat combined with the disappearance of tigers has potentially made dholes in Baluran NP more crepuscular and altered their hunting strategy to target larger prey, possibly reducing the dietary overlap with leopards. Generally, larger dhole packs tend to hunt larger prey with a high success rate (George et al., 2021), although the per capita intake appears to be less in larger packs (Bhandari et al., 2021). In Baluran NP the largest pack observed in camera trap photos and videos was 13 mature individuals. Three to four dholes have been observed to kill a Timor deer (Pudyatmoko, 2005) while nine dholes can successfully bring down a female or subadult banteng (Indrawan et al., 1996). In a recent study from India dhole pack size was found to be positively associated with prey density and negatively influenced by tiger density (Bhandari et al., 2021). Further studies are needed to decipher whether our observations of dholes alone or in pairs are temporal splits from a larger pack, newly established packs, or dispersing individuals.

### 4.5 Implications for conservation

Globally dholes are threatened by prey depletion with several of their main prey species considered vulnerable or threatened (Wolf & Ripple, 2016). However, in Baluran NP wild prey species were regularly detected in camera traps although several species have been shown to avoid the northern parts of the park due to livestock grazing (Pudyatmoko, 2017). In this study we specifically investigated temporal patterns and overlap within and between species to gain an in depth understanding of those relationships. We chose to do this because a combined spatiotemporal analysis, i.e., (Havmøller et al., 2024), would shift the focus away from the novel discoveries of different temporal patterns for dhole pack versus singles/pairs. We fully acknowledge that excluding spatial associations limits the conclusions that can be drawn.

Dholes in Baluran NP showed a moderately high overlap in activity with humans especially around sunrise. Humans were the most frequently detected of any species and recorded throughout the national park. There is potentially an effect on dholes if human presence disrupts hunting, however, it is currently unknown if dholes avoid humans and therefore are less active during the day (as is seen in many other locations). Further research is needed to determine if dholes are avoiding humans or otherwise negatively impacted by humans. If humans push dholes to be more crepuscular this potentially explains the relatively high overlap in activity between dholes and leopards. Meaning that a high human presence might force leopards and dholes to have a higher overlap and therefore compete more.

Our finding that single/pairs have pehaks later in the day indicates that they are more likely to encounter humans. If some of these lone individuals are dispersers, they are likely vulnerable as studies from African wild dogs have found high mortality rates among dispersing individuals as they come into contact with humans (Cozzi et al., 2020; Woodroffe et al., 2020). More knowledge on dhole spatiotemporal movement patterns can help us determine when they are vulnerable for better management strategies to protect them into the future.

## AUTHOR CONTRIBUTIONS

**Linnea Worsøe Havmøller:** Conceptualization (lead); data curation (equal); formal analysis (lead); funding acquisition (lead); investigation (lead); methodology (equal); project administration (equal); visualization (lead); writing – original draft (lead); writing – review and editing (lead). **Hariyawan Agung Wahyudi:** Data curation (lead); investigation (equal); methodology (equal); project administration (equal); resources (equal); software (equal); validation (equal); writing – original draft (supporting); writing – review and editing (supporting). **Mochammad Iqbal:** Data curation (equal); investigation (equal); project administration (supporting); resources (equal); software (equal). **Ventie Angelia Nawangsari:** Data curation (equal); investigation (equal); project administration (supporting); software (equal); writing – original draft (supporting); writing – review and editing (supporting). **Johan Setiawan:** Investigation (supporting); resources (equal); writing – original draft (supporting); writing – review and editing (supporting). **Desy Satya Chandradewi:** Investigation (supporting); resources (equal); writing – original draft (supporting); writing – review and editing (supporting). **Peter Rask Møller:** Conceptualization (supporting); funding acquisition (supporting); project administration (supporting); supervision (equal); writing – original draft (supporting); writing – review and editing (supporting). **Carl Træholt:** Conceptualization (supporting); project administration (supporting); resources (equal); supervision (equal); writing – original draft (supporting); writing – review and editing (supporting). **Rasmus Worsøe Havmøller:** Conceptualization (equal); formal analysis (supporting); methodology (equal); supervision (equal); writing – original draft (supporting); writing – review and editing (supporting).

## CONFLICT OF INTEREST STATEMENT

We have no conflict of interest.

## Supporting information


Video S1.

## Data Availability

Data is available on Dryad Data Repository: https://datadryad.org/stash/share/‐f2h3feY80oID9NWLg0oOkMq3EYRD2OlkyXtQAtCkAM.
