# Supplementary material for: Exploring temporal activity of dholes, their prey, and competitors in East Java, Indonesia
Source: Ecol Evol. 2024 Jul 4;14(7):e11666. doi: 10.1002/ece3.11666 (PMC11224129; doi:10.1002/ece3.11666)
Supplement: Supplementary file 1 — Video S1. [file ECE3-14-e11666-s001.zip › videoS1_legend.docx]

Camera trap videos of a pack of eight dholes harassing a mature banteng bull (Dhole_banteng_2019) and a pack of 13 dholes harassing a water buffalo at artificial water troughs (Dhole_buffalo_2019), indicating that even large packs of dholes do not pose a threat to large healthy adult bovids.
